# A Split-Wedge Anchorage for CFRP Cables: Numerical Model vs. Experimental Results

**DOI:** 10.3390/polym14132675

**Published:** 2022-06-30

**Authors:** Marco Damiani, Nicola Nisticò

**Affiliations:** Department of Structural and Geotechnical Engineering, Sapienza University of Rome, 00184 Rome, Italy; nicola.nistico@uniroma1.it

**Keywords:** prestressing systems, FRP cables, split-wedge anchorages, experimental tests, finite element analysis

## Abstract

Fiber-reinforced polymers (FRPs) are widely used within civil structural applications either for structural retrofitting or new constructions. This is due to their appreciable mechanical properties such as high stiffness and strength, resistance to environmental effects, as well low density. Through the years, such peculiarities have encouraged researchers to apply FRP cables within the design of prestressing systems, where steel cables are systematically adopted. However, the brittleness intrinsic to FRP materials necessitates additional efforts to design the anchorage devices. In fact, tendons are here subjected to stress peaks, which need to be controlled in order to prevent the premature failure of the cable. Following this goal, authors recently studied an optimized split-wedge anchorage, for 12 mm-diameter pultruded-carbon-fiber-reinforced polymer (PCFRP) tendons, adopting double-angle (DA) wedges, and compared its performance with a single-angle (SA) wedge configuration. Tensile tests were performed on 3 SA and 2 DA prototypes, respectively, through a universal testing machine: the DA configuration exploited the average cable capacity (257 kN) once, denoting a maximum efficiency. The obtained experimental results are utilized, in the framework of the present work, to calibrate contact parameters of nonlinear finite element models. The presented numerical results helped to assess benefits of the proposed configurations and the behavior of the anchorage components: the DA configuration turned out to satisfactorily avoid stress peak superpositions on the cable, with a reduction in pressure in the loading end of the cable with respect to the SA model.

## 1. Introduction

During the last few decades, the use of FRP (fiber-reinforced polymer) composite cables for prestressing systems has gained increasing attention. In fact, FRPs are characterized by high stiffness-to-weight ratios, admirable lightness, mechanical strength, and resistance to environmental agents.

FRPs are usually tailored using a wide range of materials, such as aramid, glass, or carbon, combined with thermosetting resins as epoxy, vinylester, or polyester, to obtain a product with better properties than the single components. Corresponding products are aramid-fiber-reinforced polymers (ARFPs), glass-fiber-reinforced polymers (GFRPs), and carbon-fiber-reinforced polymers (CFRPs) [1,2,3]. Recently, research also moved attention to include nanoparticles of various materials into FRPs to enhance mechanical properties, fatigue resistance, thermal properties, and flame retardancy [4,5]. This also created the opportunity to conceive reinforced polymers with sustainable natural fibres, such as cotton, banana, jute, kenaf, hemp, coir (from coconuts), and sisal (from agave) in place of synthetic ones [6].

As far as composite cables are concerned, their commercial names are Parafil^®^, Arapree, FiBRA and Technora (ARFP cables), Polystal^®^ (GFRP tendons), the carbon fiber Leadline™ and CFCC (Carbon Fiber Composite Cables) [7,8,9], and their mechanical properties are mainly linked to the type of fibers. A relatively wide range of Young modulus (E) and strength (*f_u_*) values can be found in AFRP cables: (1) Parafil^®^ -type A ropes exhibit values of E ≈ 10 GPa and *f_u_* = 0.6 GPa, that increase until type G ropes (E ≈ 126 GPa and *f_u_* = 1.9 GPa) [10]; (2) Arapree and FiBRA are characterized by E ≈ 65 GPa and *f_u_* ≈ 1.35 GPa and (3) Technora cables have moderate stiffness (E = 54 GPa), but the highest strength (*f_u_* = 2.14 GPa) [8]. The GFRP tendon Polystal^®^ [9] is characterized by E = 51 GPa and *f_u_* = 1.5 GPa, while Leadline™ (E = 147 GPa and *f_u_* = 2.6 GPa) and CFCCs (E = 137 GPa and *f_u_* = 2.1 GPa) show similar values [8]. Regarding the environmental resistance [8], FiBRA cables can reach a breaking load equal to the nominal failure load (*P_u_*) after 11 months in alkaline solution under an applied load of 0.6*P_u_*, while (1) CFCCs reach 0.93*P_u_* after 1500 days in a NaOH (0.4%) and NaCl (3.5%) solution under a load of 0.6*P_u_* and (2) Leadline™ reaches the *P_u_* after 365 days in a NaCl (5%) solution. From the point of view of fatigue resistance, AFRP Technora, FiBRA, and Arapree can undergo [8] 2.0 × 10^6^ cycles without failure under an applied load of 0.51*P_u_*, 0.50*P_u_,* and 0.40*P_u_*, respectively, within a respective load range of ±0.13*P_u_*, ±0.29*P_u_*, and ±0.15*P_u_*, Polystal^®^ [9] cables reach 0.50*P_u_* after 3.3 × 10^7^ cycles to failure under a load range of ±0.034*P_u_* and Leadline™ and CFCCs reach [8] 0.69*P_u_* after, respectively, 10 × 10^6^ cycles at ±0.08*P_u_* and 2.0 × 10^6^ at ±0.16*P_u_*. Among them, the carbon fiber cables exhibit the highest mechanical performance [11] and costs and they can be found in most civil structural engineering works, although solutions with AFRP and GFRP tendons have also been adopted [2]. However, a GFRP/CFRP hybrid cable, which inherits affordability of GFRP and the excellent mechanical properties of CFRP, has been introduced [12], but it suffers hard exposure conditions, such as high temperature and pressure, more than its raw materials [13].

Starting from the eighties, the design of new prestressed concrete bridges gave a great opportunity for pioneering applications of prestressed CFRP cables: (1) the new single-span Shinmiya Bridge (1988) in Japan was the first bridge in the world to adopt carbon fiber composite cables (CFCC) for the prestressed concrete girders, as a solution against the corrosion induced by the salinity of seasonal wind [2,14]; (2) the two-span highway prestressed-concrete Calgary Bridge in Canada, opened to traffic in 1993, has thirteen prestressed concrete girders. Among them, four were prestressed with CFCCs and two with Leadline™ rods [2]. In the early 1990s, researchers started to focus on the use of carbon fiber tendons as stays for cable-stayed bridges. Except for the full-GFRP Aberfeldy footbridge (1993, Scotland) [15], where AFRP Parafil^®^ cables were used as stays, subsequent solutions mainly involved CFRP cables [16]: (1) a combination of CFCC 7-wire tendons and indented Leadline™ rods were used for the 24 stays of the Tsukuba full-FRP pedestrian bridge (1996, Japan), supposed to be the first CFRP cable structure in the world; (2) Leadline™ cables, with different numbers of rods, were also chosen in Zhenjiang (2005, China) for the CFRP cable-stayed footbridge in Jiangsu University; (3) hybrid solutions with the choice of steel and CFRP cables can be found in the Stork Bridge (1996, Switzerland), which was the first highway bridge with CFRP cables, and the Penobscot Narrows Bridge (2006, USA), where two steel strands were replaced with CFRP strands in three selected cables. Recently, a full-GFRP cable-stayed footbridge [17] was conceived and the design included the use of eighty 12mm-diameter pultruded-CFRP cables [11] as stays.

Nonetheless, the hardest task is the conceptualization of the system to anchor the prestressed FRP cable which consists of orthotropic unidirectional materials, characterized by a lower transversal stiffness and strength, as well as brittle failure. These make it particularly challenging to pursue the design goals, which are: (1) exploiting the maximum capacity of the FRP tendons; (2) minimizing the slippage of the cable and stress concentrations on the cable portion comprised in the anchorage.

Successful solutions for anchoring FRP tendons have been bonded and split-wedge anchorages so far. Bonded anchorages consist of a hollow cylindrical steel socket, either tapered or not, filled with resin or mortar, which adheres to the element interfaces. If the anchorage is not tapered, only bonding forces between contact surfaces and the filling material oppose the cable tension, while in tapered devices, the cable is held by friction forces, consequent to normal pressure, which act at the interface between the cable and potting material. Such devices were specifically introduced for FRP tendons, due to the lower elasticity modulus of the potting grout than that of metallic wedges: the potting technology can help to reduce the magnitude of pressure at the cable interface, but anchorages need greater anchor lengths. A further improvement (Meier et al. [18,19]) considered the use of load transfer material (LTM). This material has a variable modulus of elasticity that can avoid stress peak superpositions on the cable.

Alternatively, wedged anchorage systems can be selected. These systems date back to the 20th century [20], when they were used for anchoring metallic rods or tendons in prestressed reinforced concrete. Worth mentioning are the Freyssinet and Magnel anchorages, widespread in Europe, or the Morandi and Rinaldi systems, particularly used in Italy. American anchorages, such as the Gifford Udall and Stressteel devices, stood out to be split-wedge steel anchorages. This peculiarity has been preserved within recent applications for FRP tendons. Split-wedge devices are composed of two or more wedges and a hollow steel barrel, whose inner surface is tapered. Here, a portion of cable is arranged inside the barrel and it is then blocked by wedges. The slippage of the cable is restrained by normal pressure, which is transferred to its external surface by wedges, and consequent friction forces at the interface between the cable and wedges. However, both bonded and split-wedge models highlight drawbacks and limitations. In fact, the excessive creep of potting mortar, induced by the ambient temperature, may induce a loss of performance over time, although a potted system with good creep behavior has been introduced [21], while nonoptimal wedges can cause either an excessive pinching at the loading end of the cable or the slippage of the cable.

Focusing on split-wedge systems for FRP cables, modifications to the traditional anchorages for steel tendons are requested due to their high tilting angles, usually in the order of 5–7° [22] and small anchoring lengths. The experimental tensile test [11] on a CFRP tendon blocked through a traditional anchorage highlighted the premature slippage of the cable. Improvements proposed in the literature so far mainly concerned the shape of barrel/wedge interface, that governs the magnitude and distribution of the stress components on the cable. Sayed-Ahmed and Shrive (1998) [23,24], Schmidt et al. (2011) [25], and Terrasi et al. (2011) [26] proposed a split-wedge anchorage for CFRP cables adopting a differential angle for the barrel/wedge contact surface. Such solutions satisfactorily facilitated the reduction in the wedging effect at the loaded end of the cable.

The same goal was efficiently fulfilled by the anchorage device of Al-Mayah et al. (2006) [27], who introduced a circular profile for the interface between barrel and wedges, which was proposed again by Heydarinouri et al. (2021) [28]. The efficience of curved interfaces was also employed by Gribniak et al. (2019) [29], who devised, with the help of the 3D-printing technique, a full shear-grip curved anchorage inspired by the *Nautilus* shell profile for CFRP strips.

Research efforts aimed (Damiani et al. [11]) to investigate the validity of an optimized steel anchorage, for CFRP cables, having a double-tilted surface for the wedges: experimental tensile tests highlighted the efficiency of the device.

The presented optimized split-wedge anchorage forms the core of the present paper, which is devoted to the numerical studies of the system by nonlinear finite element analyses.

## 2. Conceptualization and Review of Split-Wedge Anchorages

**Figure 1:**
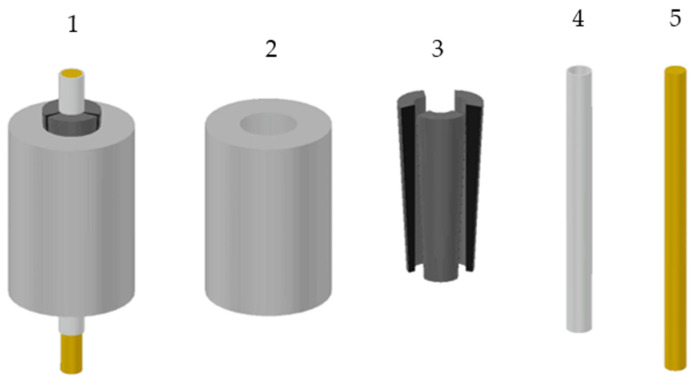
Components of a split-wedge anchorage: (**1**) 3D representation of the anchorage; (**2**) metallic barrel; (**3**) internal wedges; (**4**) sleeves; (**5**) core bar.

According to the literature, anchorages for FRP cables can be divided into bonded and mechanical, which contain the split-wedge systems. A review of bonded anchorages is provided in [11], while principles are illustrated in [30], where a hybrid bonded/split-wedge anchorage is also presented. Split-wedge anchorages are composed of (Figure 1): (1) an external tapered-steel barrel; (2) two or more wedges and (3) the FRP cable, often protected by metallic sleeves (made of aluminum or copper), which envelope the cable portions arranged inside the wedges. Sleeves also contribute to a uniform distribution of pressure.

Split-wedge systems for FRP cables descend from those traditionally used for metallic bars since the beginning of the last century. The cable is held inside wedges by the normal pressure and the consequent friction forces, which act on contact surfaces: once the cable is pulled, wedges provide a passive pressure to the cable by sliding on the tilted inner surface of the barrel, and tangential forces occur at the interface due to the friction.

**Figure 2:**
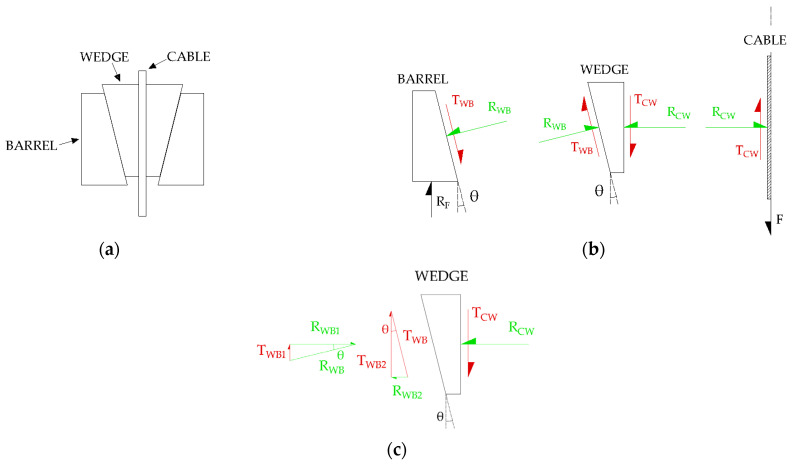
Simplified analytical model for a split-wedge anchorage: (**a**) 2D scheme of the anchorage; (**b**) Force resultants acting on interfaces of half anchorage; (**c**) Components of radial and tangential resultants on wedge interfaces.

The tilting angle and friction properties of the wedge–barrel interface play a crucial role in the radial stress distribution in the cable. Analytical simplified models available in the literature are useful to show the basic principles which govern the behavior of split-wedge anchorages. The two-dimensional static model (Figure 2a) by Campbell et al. (1997), reported in [31,32], considers the equilibrium of forces, which act on interfaces of half of the anchorage, due to the cable pull. Figure 2b details the forces at play on the interfaces. The equilibrium of friction forces on wedge (Figure 2c) holds

(1) $$T_{\mathrm{CW}}=T_{\mathrm{WB}1}+T_{\mathrm{WB}2}=R_{\mathrm{WB}}\sin\theta +T_{\mathrm{WB}}\cos\theta$$

where $T_{\mathrm{CW}}$ is the friction force between cable and wedge, $T_{\mathrm{WB}1}$ and $T_{\mathrm{WB}2}$ are the vertical tangential components of $R_{\mathrm{WB}}$ and $T_{\mathrm{WB}}$ respectively, and θ is the tilting angle of the barrel/wedge interface. Equation (1) can be rewritten as

(2) $$\frac{F}{2}=R_{\mathrm{WB}}\sin\theta \;+{\;R}_{\mathrm{WB}}\mu_{\mathrm{WB}}\cos\theta$$

Having expressed

(3) $$T_{\mathrm{WB}}=R_{\mathrm{WB}}\mu_{\mathrm{WB}}$$

and

(4) $$T_{\mathrm{CW}}=\frac{F}{2}$$

Rearranging Equation (2), the expression of the resultant force on half of the wedge-barrel is

(5) $$R_{\mathrm{WB}}=\frac{F}{2\left(\sin\theta +\mu_{\mathrm{WB}}\cos\theta \right)}$$

Knowing $R_{\mathrm{WB}}$, the normal resultant force on the cable can be analogously found as

(6) $$R_{\mathrm{CW}}=\frac{F}{2\left(\sin\theta +\mu_{\mathrm{WB}}\cos\theta \right)}\left(\cos\theta -\mu_{\mathrm{WB}}\sin\theta \right)$$

**Figure 3:**
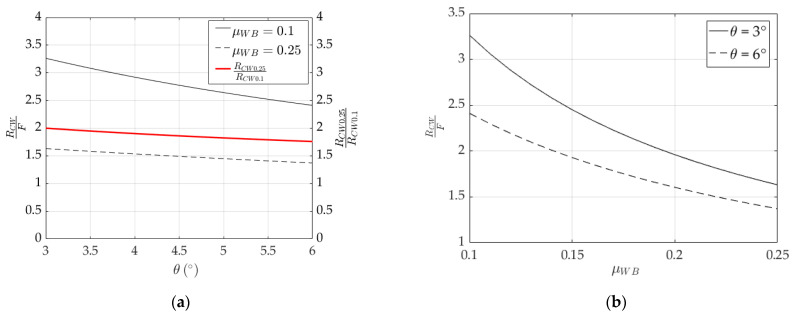
Normalized radial resultant force ($R_{\mathrm{CW}}$). Variation in: (**a**) $\frac{R_{\mathrm{CW}0.25}}{F}$, $\frac{R_{\mathrm{CW}0.1}}{F}\;$(black curves) and $\frac{R_{\mathrm{CW}0.25}}{R_{\mathrm{CW}0.1}}\;$(red curve) over the tilting angle (θ) for the two boundary values of $\mu_{\mathrm{WB}}$ (0.25 and 0.1); (**b**) Variation in $\frac{R_{\mathrm{CW}}}{F}$ over friction coefficient ($\mu_{\mathrm{WB}}$) for the two boundary values of θ (3° and 6°).

Plots of $R_{\mathrm{CW}}$ (Equation (6)) normalized over F are shown in Figure 3 by varying values of θ, within the range 3° ÷ 6°, and $\mu_{\mathrm{WB}}$, within the range 0.1 ÷ 0.25. From Figure 3a,b, it can be stated that $R_{\mathrm{CW}}$ decreases with increasing the angle (θ) and the coefficient of friction between the barrel and wedge ($\mu_{\mathrm{WB}}$), due to two reasons: (1) a greater angle would reduce the sum of the horizontal components of $R_{\mathrm{WB}1}$ and $R_{\mathrm{WB}2}$ (Figure 2c), and the magnitude of $R_{\mathrm{CW}}$ accordingly; (2) higher friction at the interface would give rise to a smaller horizontal component of $R_{\mathrm{WB}}$ [33], and consequently to a reduced $R_{\mathrm{CW}}$.

Clearly, for an efficient system, an optimal tradeoff between normal forces and friction should be adopted, based on the requested performance. Further on, other variables, such as the sleeve manufacturing, surface treatment or finishing contribute to the actual anchorage efficiency [34,35].

For the sake of completeness, it is worth mentioning other analytical models from the literature, useful to define the stress state in the cable: (1) the model of Robitaille (1999) [36], (2) the model of Persson (1964) [37], which was applied in [38], and (3) the model of Xie et al. (2015) [39].

### 2.1. Traditional Split-Wedge Anchorages

Anchorage systems for post-tensioning metallic bars have old origins. The first patent of a wedged anchorage for metallic bars was proposed at the beginning of the 20th century by Eugène Freyssinet (France). This paved the way to other concepts, later introduced all around the world, which are reported below.

#### 2.1.1. The Freyssinet Anchorages

**Figure 4:**
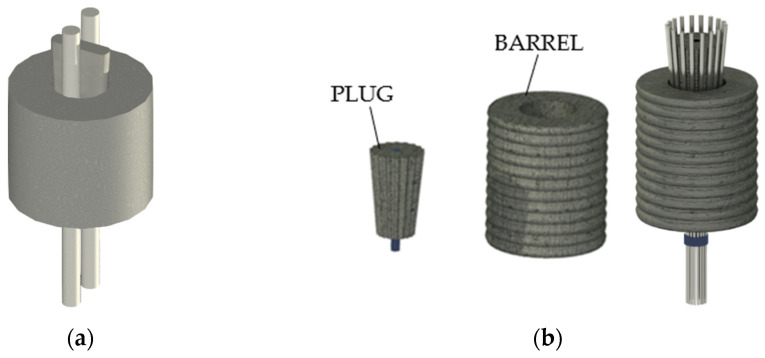
Freyssinet wedged anchorages: (**a**) Wedged anchorage for 2 rods (1907); (**b**) Concrete plug anchorage (1935) for 18 bars.

Freyssinet patented two systems [20]: (1) a wedged anchorage (Figure 4a) for two metallic rods (1907) and (2) a reinforced-concrete anchorage (1935), reported in Figure 4b, that allowed the simultaneous blockage of multiple bars (2, 3, 12, or 18). This model was composed of a grooved conical plug (with a number of notches equal to the number of rods) and a cylindrical barrel. Both parts of this device were entirely made of concrete: the barrel was reinforced with a double-spiral steel reinforcement at both the inner and outer surface. Bars were passed through the hollow cylinder and then blocked by the grooved plug.

#### 2.1.2. The Rinaldi System

**Figure 5:**
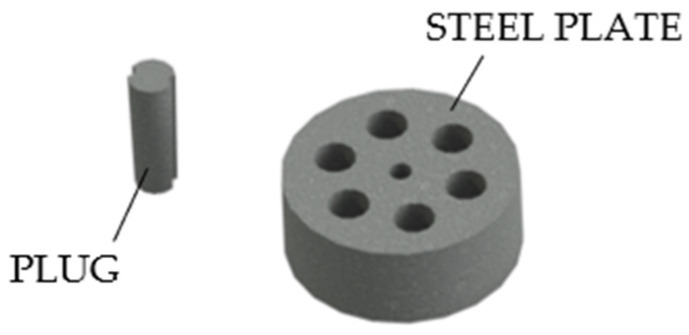
The Italian Rinaldi system.

Rinaldi (Italy), proposed [20] a system composed (Figure 5) of a circular, thick bearing steel plate with multiple tapered holes. One couple of rods were passed through each hole and then blocked by grooved steel plug.

#### 2.1.3. The Morandi System

**Figure 6:**
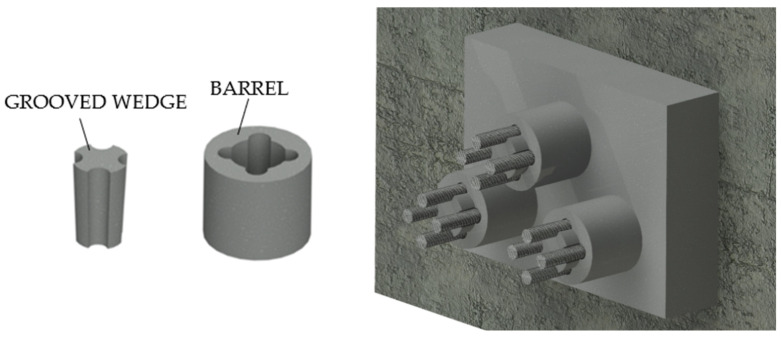
The Morandi system.

Morandi (Italy) proposed and patented [20] a system, for two steel rods, that differs from the Rinaldi system by the notched tapered holes in the bearing plate. A later enhancement introduced one more rod for each hole, with a total number of three rods. The plug was grooved with three notches here. Another model by the author was the wedged anchorage for four cables (Figure 6).

#### 2.1.4. The Magnel System

**Figure 7:**
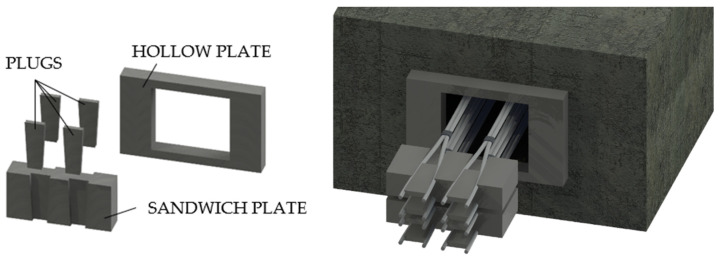
The Belgian Magnel System.

The Belgian Magnel (Figure 7) [20] anchorage was composed of a hollow bearing plate integral to the concrete, which supported other steel plates called “sandwich”. These were grooved to accommodate the steel rods, which were pulled two at a time and then blocked by steel plugs. This device allowed the realization of cables with many bars: 64-bar cables having a diameter of 7 mm each.

#### 2.1.5. The Gifford Udall System

**Figure 8:**
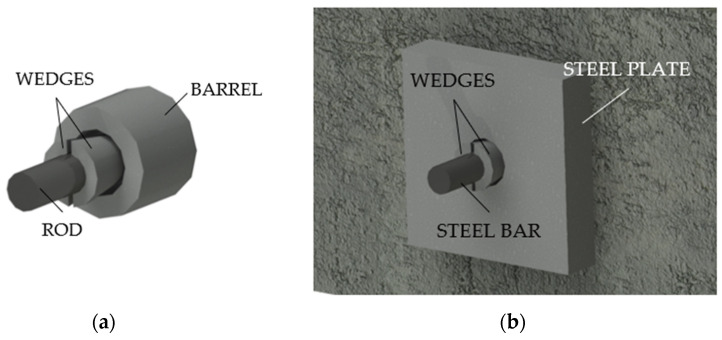
Traditional split-wedge systems: (**a**) Gifford Udall anchorage; (**b**) Stressteel Co. system.

The American Gifford Udall (Figure 8a) consists [20] of: (1) a barrel typically ≈2 cm wide and ≈2.54 cm long; (2) two half wedges with indented inner-surface and (3) rods up to a diameter of 7 mm.

#### 2.1.6. The Stressteel Co. System

The Stressteel system (Figure 8b) [20] descended from the British Lee McCall model and it was composed of two half wedges. Unlike the Gifford Udall anchorage, this device used a steel bearing plate with a tapered hole as a socket for the wedges. Moreover, the bar, having a diameter of 26 mm, was protected by a metallic sheath.

### 2.2. Optimized Split-Wedge Anchorages for FRP Cables

Split-wedge anchorages recently designed for FRP cables have preserved properties found in traditional devices, as those widespread in USA. Models proposed in the literature so far were fundamentally tailored for CFRP bars with a diameter below 10 mm. The optimized anchorage device investigated by the authors [11] uses a 12 mm-diameter CFRP bar, that could be considered a novelty.

Proposed optimized systems can be classified based on the barrel and wedge shapes that can be either straight or curved, having or not differential angles. Performance of optimized anchorage systems, moreover, should need to be experimentally and numerically validated, as reported in the scientific literature. The following subsections aim to outline aspects of the numerical modelling of anchorages by reviewing choices adopted by authors so far, as well as the results of numerical assessments.

#### 2.2.1. Differentially Angled Interfaces

**Figure 9:**
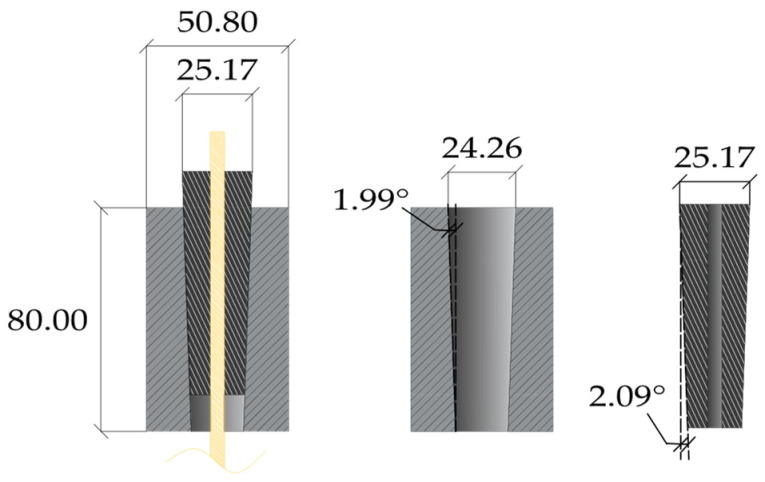
Anchorage system of Sayed-Ahmed and Shrive (dimensions in millimeters).

Sayed-Ahmed and Shrive (1998) [23] proposed a split four-wedge system (Figure 9) for ϕ = 8 mm Leadline™ cables adopting a differential angle for barrel and wedge, respectively, tilted at 1.99° and 2.09°. Results of tensile tests highlighted a maximum failure load of 124 kN, which was higher than the nominal ultimate load of the cable. Regarding the fatigue strength, the system could undergo a maximum number of cycles equal to 2.42 × 10^6^. A finite element model was implemented in order to assess the stress distribution along the FRP cable. Eight-node isoparametric elements were used for the anchorage and the cable, while interface elements were used along the contact surface lines. Values of coefficients of friction equal to 0.5 and 0.05 were adopted for wedge/cable and barrel/wedge contacts, respectively. Two different models were implemented: (1) a linear model, considering linear elastic materials for each component, and (2) a nonlinear model, introducing the plastic behavior of the steel parts. Analyses were carried out in three load steps: (1) simulation of the wedge seating by applying a displacement on the top surface; (2) release of the applied displacement; (3) application of the tensile force to the CFRP cable. Results of nonlinear analyses at the end of the third step highlighted that all the stress components (radial, shear, and longitudinal) had peak values at the loading end of the cable, while linear analyses returned stress profiles with peaks at different locations. Campbell et al. (2000) [31] implemented a finite element model of the anchorage reported in [23], adopting linear elastic materials, to assess the influence of (1) different values of coefficient of friction (0.05, 0.1, 0.2, and 0.3) and (2) differential angles between barrel and wedge (0, 0.06, 0.11, and 0.2) on the stress distribution along the tendon, assuming a coefficient of friction between wedge and CFRP tendon equal to 0.4 and without providing any preset load. The main results were: (1) radial stress in the cable increases with decreasing the coefficient of friction. Clearly, lower friction requires higher normal pressure to ensure the equilibrium at the same level of tensile force. (2) Adopting coefficients of friction of 0.2 and 0.4 for barrel–wedge and wedge–tendon contacts, respectively, a differential angle of 0° returns a radial stress distribution, which reaches a value of 220 MPa. With increasing the differential angle, the free end of the cable tends to unload, until reaching zero radial stress for a value of 0.2°, which means that no contact exists between the barrel and wedge.

**Figure 10:**
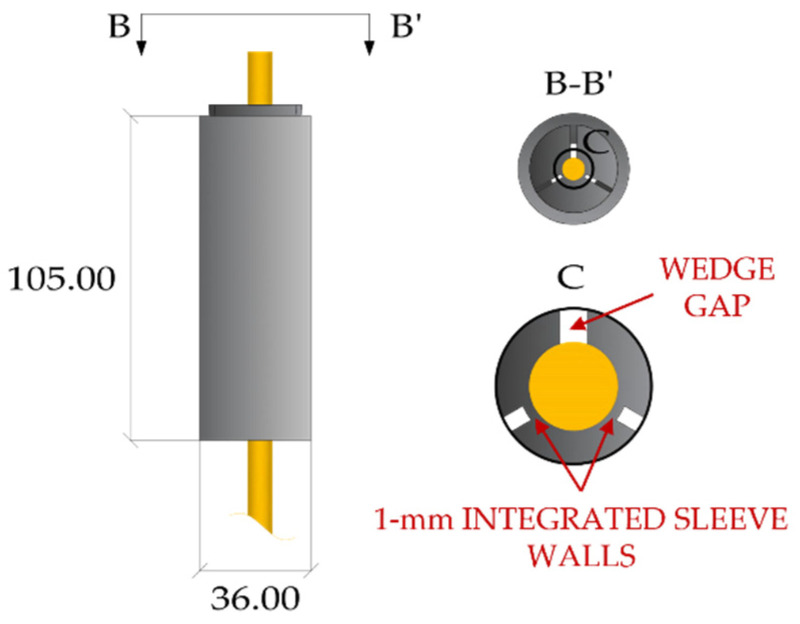
Split-wedge anchorage of Schmidt et al. (dimensions in millimeters).

Schmidt et al. (2010) [25] introduced a split three-wedge anchorage (Figure 10) for ϕ = 8 mm CFRP rods characterized by a unique sleeve-wedge element, having a differential angle of 0.4° with the inner surface of barrel (tilted at 3°). The aluminum sleeve-wedge part was obtained by notching three radial slits on the wedge body: (1) one fully separates two wedges, giving rise to a gap; (2) the other two slits leave 1 mm walls, in contact with the rod, which connect the three wedges. Such solution can maximize the gripping surface and provide a more uniform radial stress distribution around the cable surface. Tensile tests performed on five specimens returned failure loads ranging from 142 kN to 149 kN, which turned out to be greater than the manufacturer’s mean value (120 kN). Schmidt et al. (2011) [40] numerically simulated, with the Abaqus software [41], the anchorage through a nonlinear 3D finite element model. Hexahedral elements were used to discretize both the CFRP rod and the barrel, and tetrahedral elements for the sleeve-wedge system. The FEM model accounted for the plastic behavior of the barrel and wedges and anisotropic elastic properties of the CFRP rod. The barrel/wedge and wedge/rod interfaces were modeled with a surface-to-surface discretization, adopting a finite sliding formulation [41] and a penalty friction [42]. Finite element analyses were performed on one half of the model, due to the symmetry, and results in terms of circumferential strains on the outer surface of the barrel were compared with the experimental ones, elaborated by an ARAMIS 3D optical measurement system. Strain profiles highlighted maximum values at the wedge gap. Moreover, variation in the transverse elastic modulus of the rod from 2000 MPa to 7600 MPa did not seem to greatly afflict strains at the slit and gap, except for the barrel surface comprised in between. Contact pressure on the CFRP rod also exhibited greater values at the unloaded end of the anchorage and magnitudes close to zero at the loaded end.

**Figure 11:**
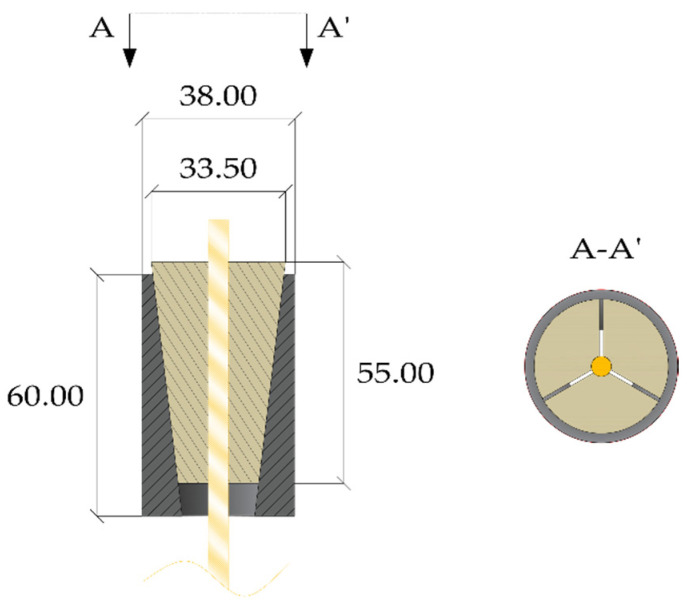
Split-wedge anchorage of Terrasi et al. (dimensions in millimeters).

The thermoplastic polyphenylene sulfide (PPS) polymer wedges were adopted in conjunction with a sand-coated CFRP tendon (ϕ = 5.4 mm) by Terrasi et al. (2011) [26] (Figure 11) to design a split-wedge anchorage. A first model was subjected to static tensile tests, highlighting an average failure strength 58.7% less than the tendon’s tensile strength, equal to 2000 MPa. Then, an optimized model was designed by adopting: (1) a differential angle of 0.23° between the barrel and wedge; (2) a longer wedge and barrel and (3) local modifications (chamfers). Tensile tests on the optimized anchorage showed an average failure strength 25% greater than the first system. Abaqus finite element analyses [41] were utilized to assess the stress distribution on the CFRP rod and to perform the design optimization. A 3D finite element model of one sixth of the anchorage was implemented due to the symmetry, and simulations were performed by applying a tensile stress of 1000 MPa to the cable. Contacts at interfaces were defined through the node-to-surface formulation: (1) contact between the sand-coated rod and wedge was modeled as soft contact, assigning a user-defined constitutive curve iteratively defined based on a compression test result on the rod; (2) a low friction coefficient was assigned to the barrel–wedge interface due to the application of lubricant. Finite element results showed a better performance with respect of the unoptimized system: (1) radial stress, albeit preserving a similar magnitude, had the peak value moved to the unloaded end of the cable; (2) shear stress on the cable surface, having a flatter distribution, denoted a reduction of the 25% and (3) cable axial stress was 10% lower.

#### 2.2.2. Curved Interfaces

**Figure 12:**
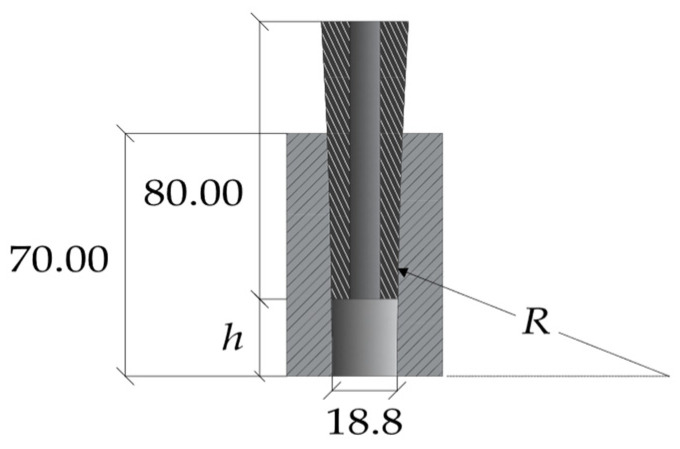
Curved anchorage of Al-Mayah et al. (dimensions in millimeters).

Al-Mayah et al. (2006) [27] introduced a split four-wedge steel anchorage (Figure 12) for CFRP cables characterized by a circular profile between the inner surface of the barrel and the external surface of wedges, that were shaped with the same radius. Experimental tests were performed by varying cable diameters (ϕ = 6.4 mm and ϕ = 9.4 mm), the seating distance of wedges from the loading end of the system, and the radius of the circular interface. Results showed that: (1) higher values of the radius increase the displacement of the rod and (2) no premature failure occurred. Authors also performed finite element analyses in order to determine the stress state inside the anchorage [43]. A first 3D nonlinear model was implemented using eight-node linear brick elements for the components, except for the inner layer of the rod, that was modeled through six-node triangular elements. Materials were considered linear elastic and the friction coefficients adopted were: (1) 0.0 ÷ 0.02 for the barrel/wedge interface, due to the lubrication; (2) 0.4 for the sleeve/wedge interface and (3) 0.24 for the sleeve/rod interface, obtained by experimental pull-out tests. The results highlighted that: (1) the radial stress peak are located near the free end of the cable; (2) as the radius increases, the contact pressure decreases along the rod length, approximately maintaining the same profile.

**Figure 13:**
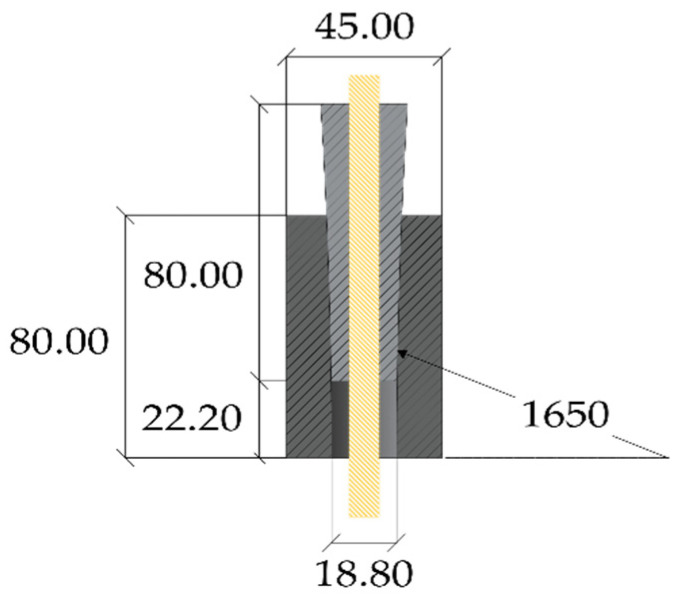
Curved anchorage of Heydarinouri et al. (dimensions in millimeters).

Heydarinouri et al. (2021), similarly to [27], proposed and tested [28] a curved split-wedge anchorage (Figure 13) for CFRP rods (ϕ = 8 mm), but with aluminum wedges and removal of the sleeve between the cable and wedges. Tensile tests on the system returned a breaking load 16% greater than the cable ultimate load, while fatigue tests highlighted that no rupture occurred within 2.0 × 10^6^ cycles, although slippage between the wedges and cable occurred in some specimens. The proposed system was numerically modeled [44] in the Abaqus software [41] and parametric analyses were carried out considering: (1) differential angles, between barrel and wedges, of 0.1°, 0.16°, and 0.23°; (2) different fillets (circular and straight) at the tip of wedges for a differential angle equal to 0.1°. Materials were treated as linear elastic, except for the aluminum of wedges, which was provided with plastic behavior. Contact surfaces were modeled through a surface-to-surface discretization, with the finite sliding formulation. A “hard contact” behavior [41] was assigned to the normal behavior, while the penalty formulation [42] was adopted for the tangential behavior, adopting a coefficient of friction equal to 0.19 and 0.3 for the wedge–barrel and rod–wedge interfaces, respectively. The main results were: (1) by increasing the differential angle from 0.1° to 0.23°, the peak value of contact pressure decreases at the loading end of the CFRP cable; (2) the modified anchor, either with round (radius of 4 mm) or straight fillets (cut angle of 40°), exhibited a reduced contact pressure at the tip of the wedges; (3) the Tsai–Wu failure criterion [45] was adopted to establish the optimum design among the proposed models and the Tsai–Wu failure index was calculated based on the stress state of the CFRP cable. The maximum index (2.25) was found in the model with constant differential angle equal to 0.1°, but fillets could reduce it to 1.37. Curved anchorage showed the minimum failure index, and it was thus chosen as the optimal design.

In conclusion, the works regarding anchorage models for FRP cables proposed so far aim to mitigate the stress state in the cable, whilst exhibiting high efficiency, which is defined as the ratio between the system and the cable capacity. Cables with a diameter within 8 mm have been mainly adopted and stress states obtained through numerical analyses generally cannot be extended to the system investigated here.

Thus, the present work aims to provide a specific definition of the contact relationships at interfaces, based on the performed experimental tests on the anchorages for ϕ = 12 mm cables.

## 3. The Optimized Double-Angle Split-Wedge Anchorage

**Figure 14:**
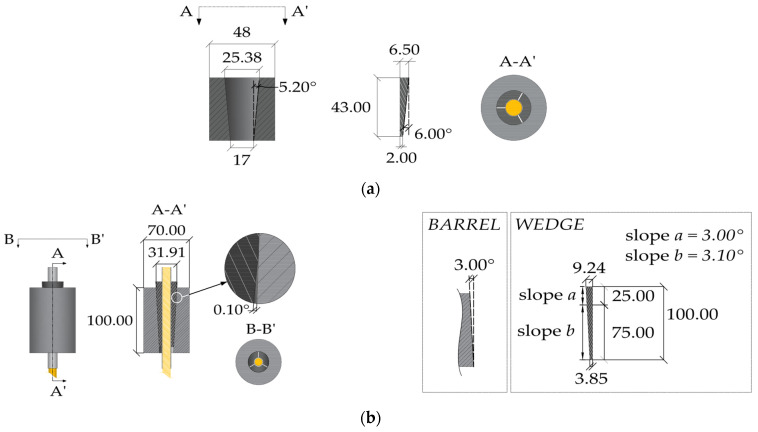
Geometry of the anchorage models tested within the experimental campaign (dimensions in millimeters): (**a**) Traditional anchorage model; (**b**) Optimized double-angle wedge model.

Traditional anchorages for steel cables are usually conceived with differential angles between the barrel and wedges. An example, for a 0.5 inch cable, is reported in Figure 14a: (1) the contact surface between barrel and wedges, both made of steel, is smooth; (2) angles are equal to 5.2° (inner barrel surface) and 6° (external wedge surface). As previously reported, anchorages with differential angles have been also designed for CFRP cables so far: (1) a differential angle of 0.1° (Figure 9) has been assumed in [23], where the barrel and wedge are tilted at 1.99° and 2.09°; (2) a differential angle of 0.4° (Figure 10) was proposed in [25], where the inner surface of the barrel is tilted at 3°; (3) a differential angle of 0.23° (Figure 11) was adopted in [26]; (4) a curved anchorage (Figure 13) was introduced in [27] and also presented in [28], where numerical analyses highlighted that, with increasing the differential angle from 0.1° to 0.23°, the peak value of contact pressure at the loaded end of the CFRP cable decreases.

Further on, the authors investigated [11] two solutions of a split-wedge steel anchorage, whose geometry (Figure 14b) was conceived and optimized [46] through preliminary finite element analyses, which aimed to predict global results. The selected cables were made of pultruded CFRP (PCFRP) produced by CARBONVENETA: (1) the diameter is 12 mm; (2) the mean elastic modulus along the fiber direction is 164 GPa, evaluated by the manufacturer according to the ISO 10406-1:2015 standards [47]; (3) the mean nominal axial strength is equal to 2275 MPa; (4) the cable surface was treated to improve the grip. Further on, the portions included into wedges were protected by two 1mm-thick aluminum sheaths, with a length of ≈15 mm, glued to the cable through Sika ADEKIT H9952 BK [48] epoxy resin. The steel parts were composed of: (1) a 100 mm-long steel barrel with inner surface tilted at 3°; (2) three 100 mm wedges, assuming two different configurations, denoted as single angle (SA) and double angle (DA). A constant angle of 3° was adopted in the SA solution, while in the DA solution, 25% (25 mm) of the external surfaces of wedges was tilted at 3° and the remaining 75% (75 mm) at 3.1°, giving rise to a differential angle of 0.1° with the barrel. Steels adopted for the barrel and wedges and properties provided by the producer were, respectively: (1) C45 (E = 220 GPa; *f_y_* = 395 MPa; *f_u_* = 649 MPa); (2) 16CrNi4Pb (E = 220 GPa; *f_y_* = 667.8 MPa; *f_u_* = 694.3 MPa).

The aluminum sheath and resin were subjected to tensile tests in order to obtain the main mechanical properties for the numerical models, while traditional and the SA and DA anchorages were both experimentally tested [11] and numerically analyzed. The test methods are reported in the following subsection, together with the numerical models proposed here.

### 3.1. Test Methods

**Figure 15:**
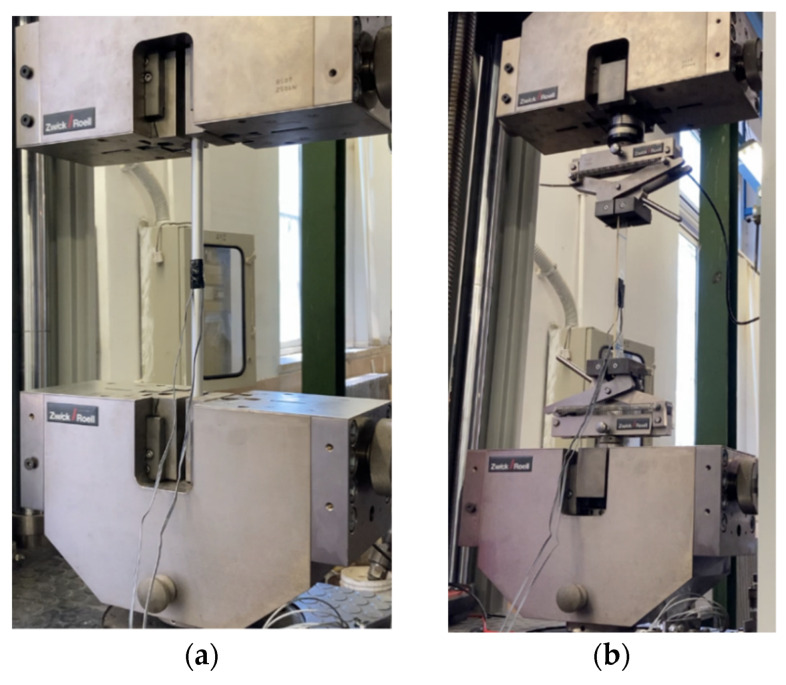
Experimental tensile tests: (**a**) Setup for the aluminum sleeve; (**b**) Setup for the epoxy resin stripe.

**Figure 16:**
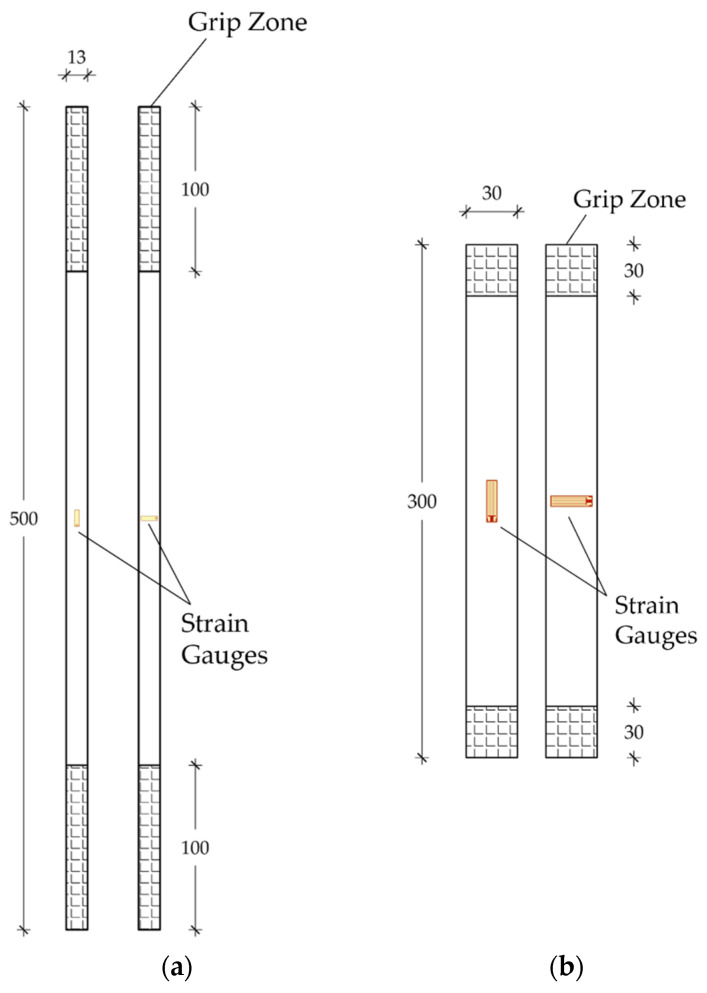
Experimental tensile tests: (**a**) Two sides of the sleeve specimen; (**b**) The two sides of the resin specimen.

Specimens were subjected to tensile tests at the laboratory of the Department of Structural and Geotechnical Engineering (Sapienza University of Rome). Specifically, one specimen of the aluminum sleeve (h = 50 cm) and a rectangular strip (h = 30 cm, w = 3 cm, and t = 0.4 cm) of resin were first tested (Figure 15a,b and Figure 16a,b) through a Zwick Roell testing machine. Longitudinal and transversal strains were acquired by means of a pair of 6 mm strain gauges (Tokyo Sokki Kenkyujo Co., Ltd., Tokyo, Japan) applied at the center of the specimens (Figure 16a,b) for each side, in the case of the resin strip. As far as the aluminum test is concerned, a force-controlled procedure was implemented with a constant rate of 2 kN/min and the two ends included inside the clamps were reinforced by two pieces of PCFRP cable, as long as the clamps (l = 10 cm), glued with the epoxy resin inside the inner hole. A tensile test on the resin sheet was performed, on the other hand, by applying a constant displacement rate of 4 mm/min.

Tests on the anchorages were carried out on 1 traditional and 5 optimized (3 SA and 2 DA) specimens through a MTS testing machine. A displacement-controlled procedure was adopted by setting a constant displacement rate of 4 mm/min and, moreover, without assigning a presetting load.

**Figure 17:**
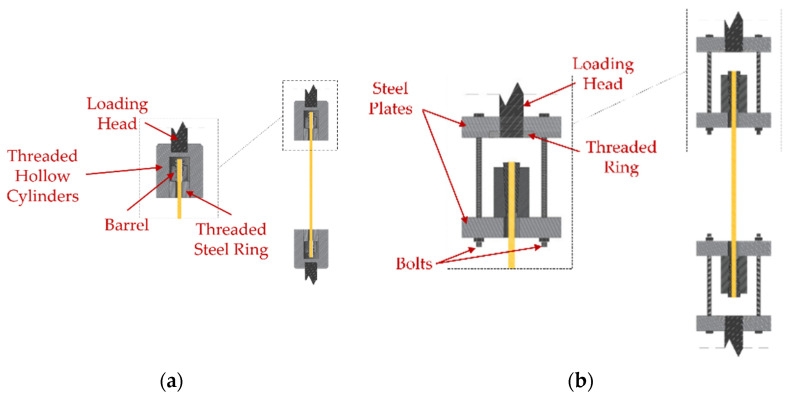
Experimental tensile tests: (**a**) Setup for the traditional anchorage; (**b**) Setup for the optimized system.

In the traditional anchorage, top and bottom barrels were placed on a threaded bearing ring and then encased into two hollow steel cylinders (Figure 17a) following two stages: (1) the bearing ring was first screwed to the cylinder; (2) the hollow cylinder was then screwed to the loading head of the MTS machine. Here, force and displacements were acquired by the MTS machine.

The setup of the new optimized anchorage specimens (Figure 17b) was composed of: (1) two pairs of perforated steel plates, with thickness of 40 mm, connected to each other through two pairs of four high-strength bolts (ϕ = 16 mm). The outer plates at the top and bottom anchorages were first passed through the threaded machine heads and then fastened by two threaded rings; (2) CFRP cables, with a length of 600 mm, inserted throughout the plate holes and then fastened through the anchorages.

**Figure 18:**
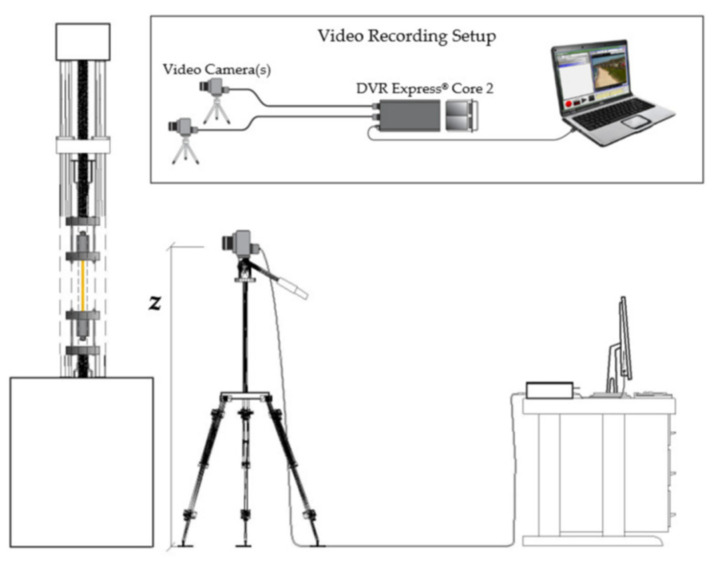
Experimental tensile tests. Setup of the DIC acquiring system.

Displacements of the five optimized anchorages (SA and DA) were obtained through a digital image correlation (DIC) code [49] by an “IO Industries” system that includes a camera “FLARE” and a digital video recorder “DVR Express^®^ Core 2”, according to the setup in Figure 18. Visible parts of the five tested specimens, to be tracked by the DIC software, were first randomly speckled. The monitored parts were: (1) the free length of the cable comprised between the two steel plates for one test and (2) the outermost part of the visible top wedge for the other four tests.

### 3.2. Numerical Models

**Figure 19:**
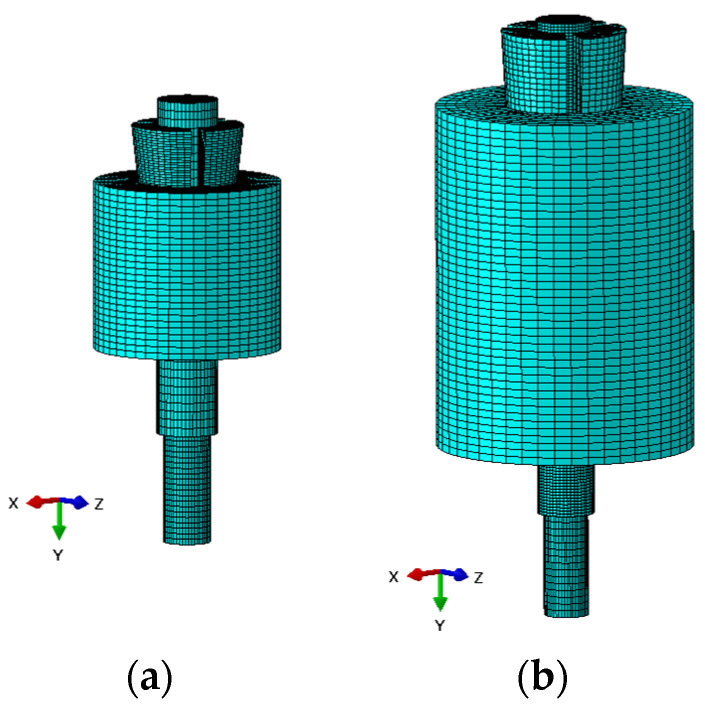
FEM model: (**a**) Traditional anchorage; (**b**) Optimized anchorage.

**Figure 20:**
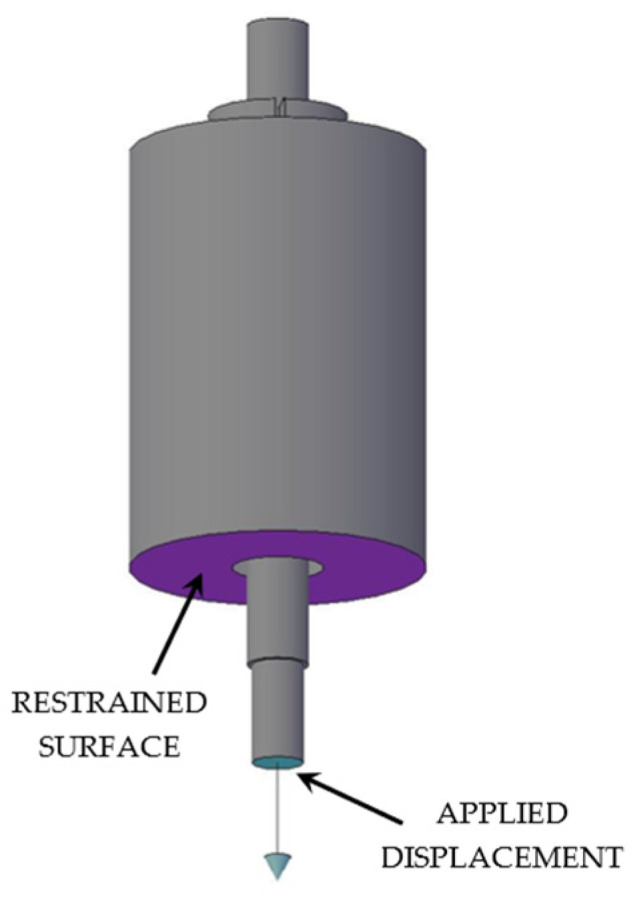
FEM model. Boundary conditions.

Numerical analyses of the anchorage presented here were performed through the finite element method (FEM), using the software Abaqus [41]. Traditional, SA and DA anchorages were modeled (Figure 19a,b) through C3D8R elastic brick elements having isotropic material for all the subsystems, with exception of the cable considered orthotropic. The testing load was simulated by imposing a fixed displacement to the cable end, having restrained the barrel bottom surface (Figure 20).

**Figure 21:**
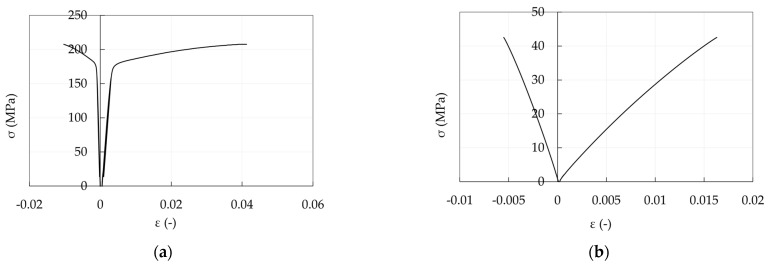
Experimental stress-strain curves: (**a**) Aluminum sleeve; (**b**) Epoxy resin.

**Figure 22:**
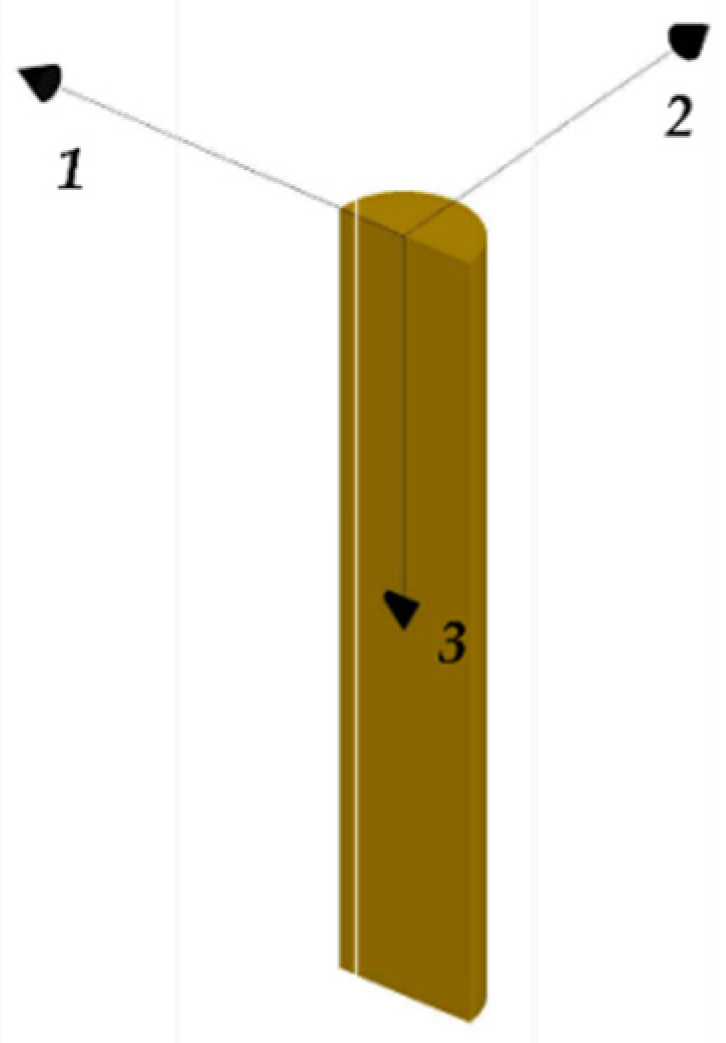
FEM model. Material reference system assumed for the CFRP cable.

Young moduli (E, GPa) and Poisson coefficients (*ν*) of anchorage materials adopted in the analysis are shown in Table 1 and Table 2: (1) steel properties of barrel (C45) and wedges (16CrNi4Pb) refer to the producer values; (2) aluminum properties are the design values provided by the Eurocode 9 [50] (E = 70 GPa and *ν* = 0.3). Such properties can be obtained from a stress level equal to the 25% and 50% of the tensile strength in Figure 21a, respectively; (3) resin properties were experimentally evaluated (Figure 21b) and they are extracted from a stress level of 25% of the tensile strength; (4) PCFRP properties are those provided by the producer and the assumed material directions pertain to the reference system in Figure 22.

**Figure 23:**
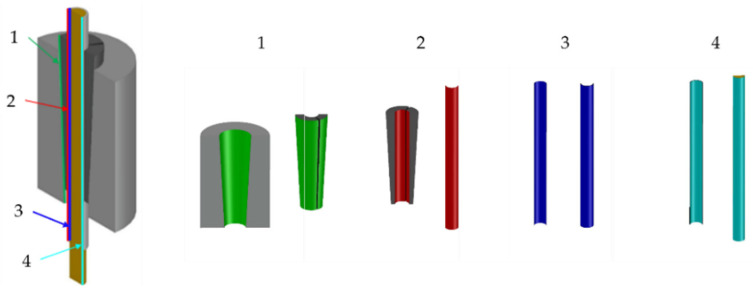
FEM model. Contact surfaces: (**1**) barrel/wedges (green); (**2**) wedge/sleeve (red); (**3**) sleeve/resin (blue); (**4**) resin/cable (cyan).

Contacting part surfaces (Figure 23) were connected to each other by means of surface-to-surface contacts.

A *hard* contact is defined along the normal direction for all the contact pairs, with the exception of the barrel/wedge interface. Here, a *soft* contact is assumed imposing a relationship between the acting pressure and the current overclosure. Both the hard contact and Coulomb friction behavior have been enforced through the penalty method [42], which allows a small amount of both penetration along the normal direction and tangential displacement, in the stick condition, before the attainment of the critical Coulomb shear stress.

Slips are neglected when a specific option, named *rough*, is adopted based on the experimental evidence. Relative motion between two paired surfaces has been evaluated through the finite-sliding tracking approach [41]. Forces occurring between two paired surfaces are split according to: (1) tangential and (2) normal directions.

**Figure 24:**
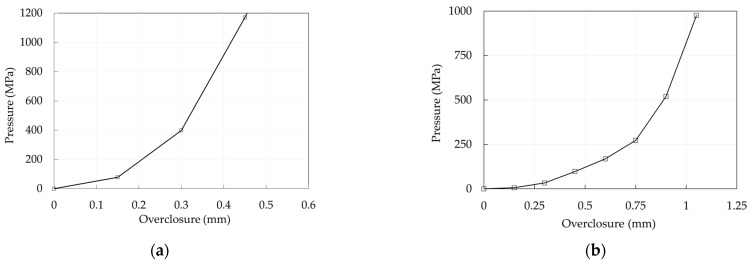
Anchorage models. Adopted pressure-overclosure relationships for the barrel/wedge interfaces: (**a**) Traditional anchorage; (**b**) Optimized anchorage.

The adopted strategies are summarized in Table 3 for the traditional and optimized anchorages, respectively, while the adopted pressure/overclosure relationship is reported in Figure 24a,b: it is worth noticing that these curves are consequent to a numerical investigation aimed to minimize the differences between numerical and experimental results.

## 4. Results and Discussion

Experimental results are reported in the following section, together with the numerical analyses proposed here, which aim to support the advantages of the optimized SA and DA solutions. Finite element models were calibrated through the experimental curves, and numerical results, expressed in terms of stresses on the preset contact surfaces, allowed the evaluation of benefits related to the two design strategies (SA an DA).

### 4.1. Experimental Results

**Figure 25:**
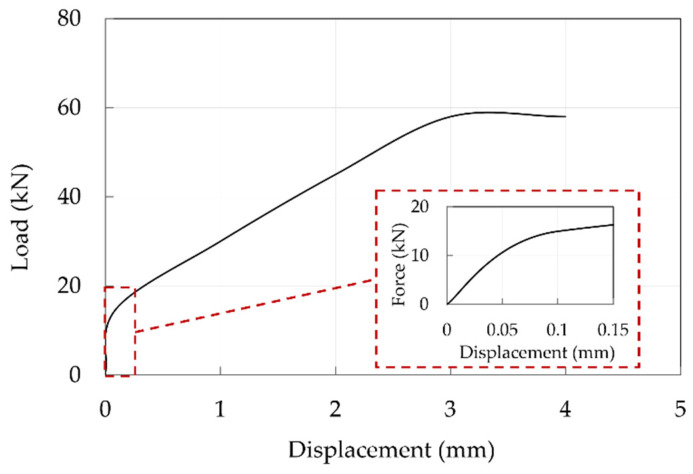
Experimental force-displacement curve of the traditional anchorage sample. The red dotted square contains a magnification within a displacement of 0.15 mm.

Global results are reported in terms of the force-displacement curves. As far as traditional anchorage is concerned (Figure 25), one specimen was subjected to the test and displacements cannot be considered reliable being recorded through the MTS system, but it can be noted that slip occurs at a load of approximately 60 kN, that is about the 20% of the CFRP cable tensile capacity. A subsequent check highlighted that relative displacements between the cable and sleeve occurred.

**Figure 26:**
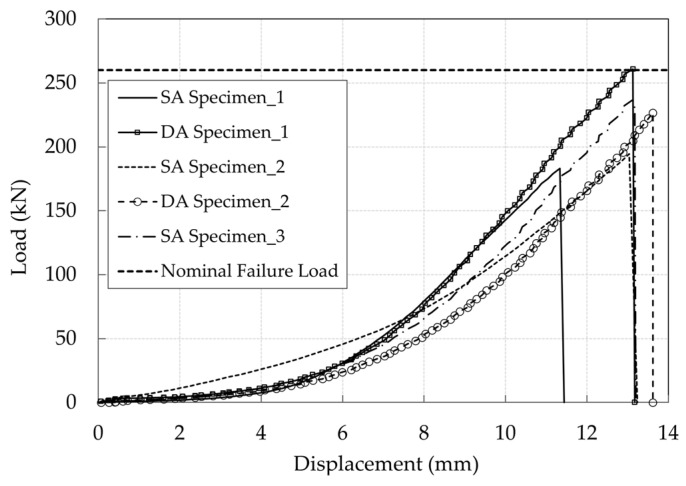
Experimental force-displacement curves of the five tested optimized anchorages elaborated by the DIC code. The cable free length was monitored in the SA Specimen_3, and the outermost part of the top wedge in the others.

Experimental results of the SA and DA solutions are reported in Figure 26. The SA wedge systems returned a failure load equal to 183 kN, 194 kN, and 232 kN respectively, with an average of 206 kN. The DA anchorages failed at 257 and 222 kN respectively, with an average failure load of ≈240 kN, that is about 15% greater than that of the SA. On the other hand, the maximum displacements of five tests are between 13.6 and 13.6 mm, except for the first test of the single-angle wedge specimen, that shows a maximum displacement of 11.4 mm. One cable reached the average nominal failure load declared by the manufacturer (257 kN), only with the DA solution, that corresponds to an efficiency of 100%.

### 4.2. Numerical Results

Results of the finite element analyses are presented regarding traditional and optimized anchorages, in terms of global and local results, that concern: (1) force-displacement relationships, where displacements are applied at the cable free end (Figure 20); (2) pressure and equivalent contact shear on the interfaces, where the latter is computed as

(7) $$\tau_{\mathrm{eq}}=\sqrt{\tau_{1}^{2}+\tau_{2}^{2}}$$

where $\tau_{1}$ and $\tau_{2}$ are the two shear components acting along the local planar directions of the contact surface.

**Figure 27:**
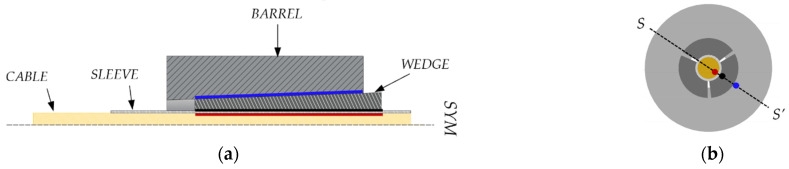
Contact surfaces. Adopted alignments for contact stresses: (**a**) Cable (red), sleeve (black), and wedge (blue); (**b**) Location of alignments along the line S-S’.

**Figure 28:**
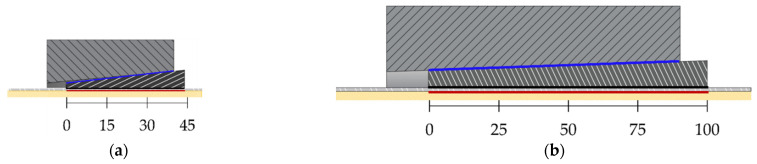
Contact surfaces. Location of reference systems: (**a**) Traditional anchorage; (**b**) Optimized anchorage.

Results of contact stresses refer to the alignments (Figure 27a) resin/cable (red), wedge/sleeve (black), and barrel/wedge (blue). Those alignments lie in a plane passing through line S-S’, as illustrated in Figure 27b. Local results along the considered alignments refer to the coordinate system reported in Figure 28a (traditional anchorage) and Figure 28b (optimized anchorage): for both systems, the origin is assumed at the sharpest end of the wedge.

#### 4.2.1. Traditional Anchorage

**Figure 29:**
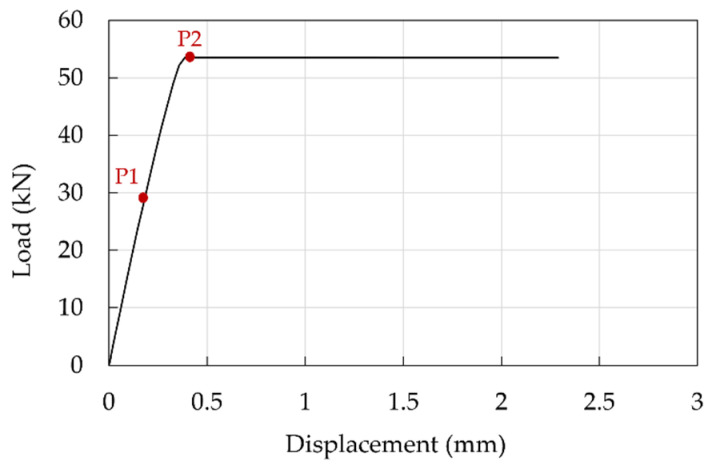
Traditional anchorage. Force-displacement curve: numerical results.

The pressure-overclosure relationship (Figure 24a) was calibrated assuming μ = 0.25 at the barrel/wedge interface and μ = 0.7 [51] at the resin/cable interface: the goal was to obtain the cable slippage at a force of ≈58 kN, in agreement with the experimental evidence. The obtained force displacement curve is reported in Figure 29 which, compared with the experimental one (Figure 25), denotes a good agreement in terms of peak force, having reached 92% of the experimental value. The following can be observed: (1) an initial linear branch, within point P2, where the cable in the stick phase experiences small displacements, without slippage, due to the penalty method and the allowed penetration of wedges into the barrel; (2) the activation of slip in the cable at point P2, after which the numerical displacements are lower than those, not reliable, recorded through the testing machine.

Information about the state of interfaces is provided by evaluating, at points P2 and P1, the stress-resultant ratios ($\frac{F_{\mathrm{shear}}}{F_{\mathrm{pressure}}}$, Equation (8)), to check the activation of the slippage according the Coulomb law and considering the friction coefficients (μ) reported in Table 3.

(8) $$\frac{F_{\mathrm{shear}}}{F_{\mathrm{pressure}}}=\mu \;$$

**Figure 30:**
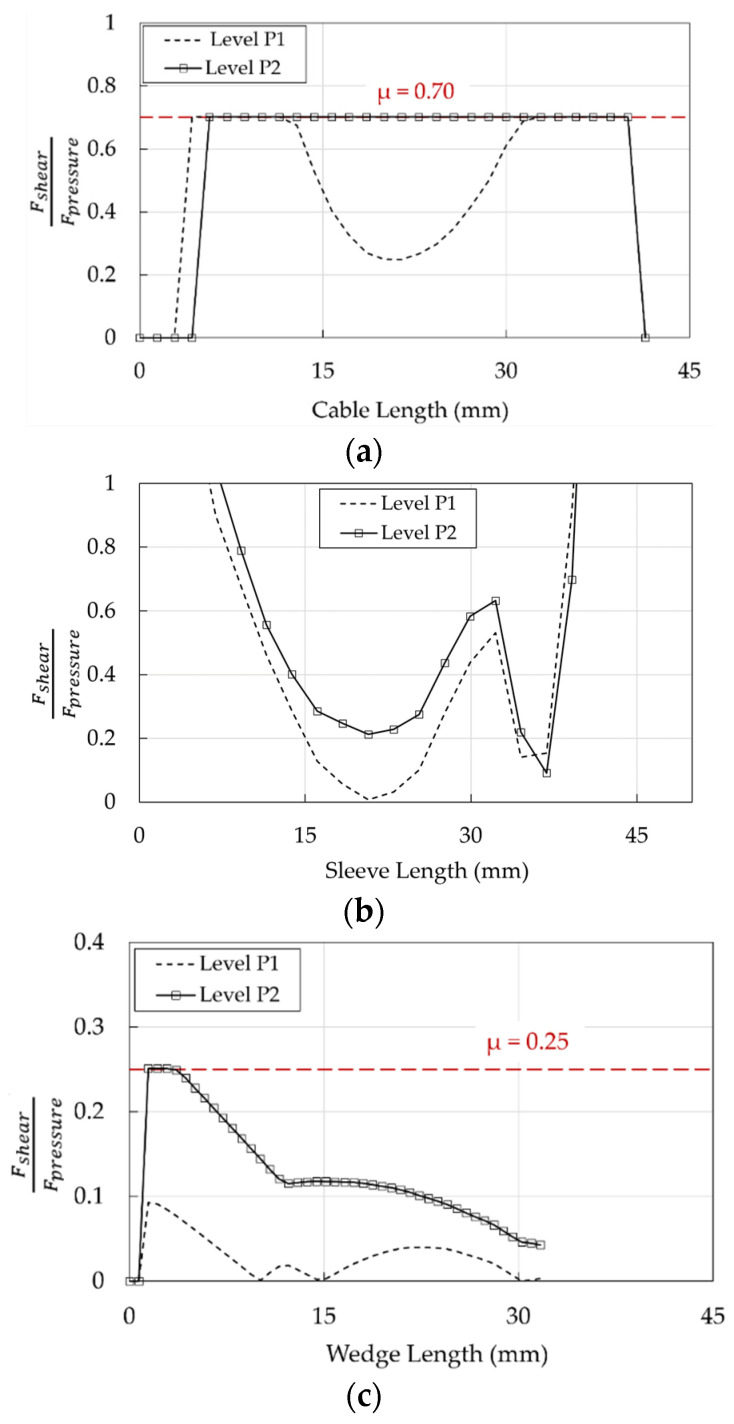
Traditional anchorage. Profiles of $\frac{F_{\mathrm{shear}}}{F_{\mathrm{pressure}}}$ at levels P1 and P2: (**a**) Cable/resin interface; (**b**) Wedge/sleeve interface; (**c**) Barrel/wedge interface.

At point P1, the interfaces resin/cable (for about half of the length) and barrel/wedge (everywhere) exhibit (Figure 30a,c) a ratio lower than the corresponding μ: this indicates that elements do not slip. From level P2, $\frac{F_{\mathrm{shear}}}{F_{\mathrm{pressure}}}$ remains below 0.25 almost everywhere in the barrel/wedge interface (Figure 30c), while it reaches 0.70 along the whole cable length in contact with the wedge (Figure 30a) and the cable starts to slip. Ratios at the wedge/sleeve interface (Figure 30b) reach high local values, as a consequence of the rough friction behavior, where no restrictions (infinite coefficient of friction) are given to $F_{\mathrm{shear}}$ in Equation (8). However, it has to be noticed that $\frac{F_{\mathrm{shear}}}{F_{\mathrm{pressure}}}$ assumes an average value of ≈0.5, which can be considered a lower bound for the friction coefficient (μ), due to the absence of sliding emerged from the experimental results.

**Figure 31:**
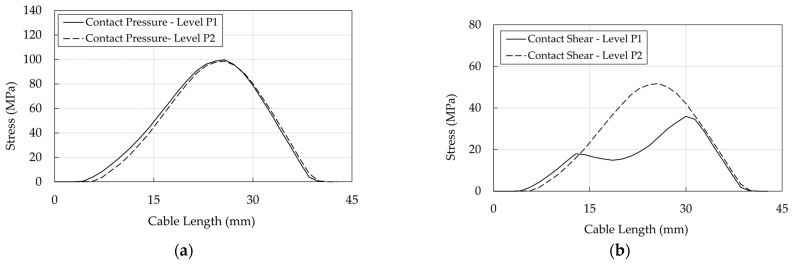
Traditional anchorage. Stress in the resin/cable interface: (**a**) Contact pressure; (**b**) Shear stress.

**Figure 32:**
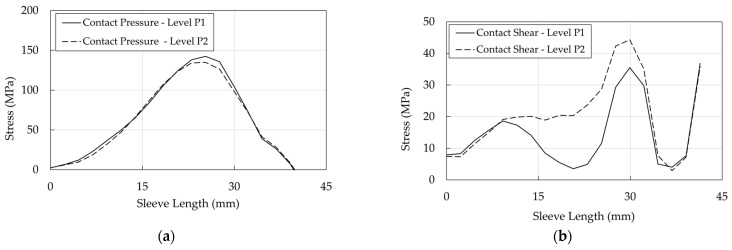
Traditional anchorage. Stress in the wedge/sleeve interface: (**a**) Contact pressure; (**b**) Shear stress.

**Figure 33:**
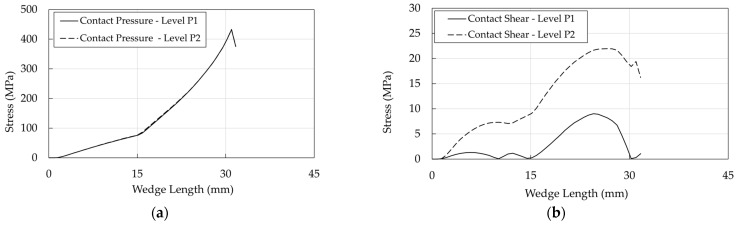
Traditional anchorage. Stress in the barrel/wedge interface: (**a**) Contact pressure; (**b**) Shear stress.

Profiles of pressure and shear at interfaces are shown (Figure 31, Figure 32 and Figure 33) at levels P1 (stick phase) and P2 (beginning of the slip phase).

Regarding the resin/cable interface: (1) stresses assume peak values (Level P2) at ≈25 mm, where contact pressure (Figure 31a) is ≈100 MPa and the equivalent shear (Figure 31b) is ≈50 MPa; (2) at level P1 (stick phase), shear exhibits (a) two peaks at ≈13 mm and 30 mm, where (Figure 30a) $\frac{F_{\mathrm{shear}}}{F_{\mathrm{pressure}}}$ is lower than 0.7, that is the assumed friction coefficient, and (b) a minimum at ≈20 mm, where $\frac{F_{\mathrm{shear}}}{F_{\mathrm{pressure}}}$ shows the lowest value (Figure 30a); (3) passing from the state P1 to P2, (a) the contact pressure does not increase with the force increase, and (b) the equivalent shear increases due to the assumed elastic behavior of the contact element.

The contact pressures of the wedge/sleeve interface (Figure 32a), considering that a hard contact has been assumed, are similar to those obtained for the the resin/cable interface. Further on, it can be observed that contact and shear stresses are higher and lower than values in the cable/resin interface, respectively, so that the slip phase does not occur, assuming that μ is at least equal to 0.7 due to roughness of the internal wedge surface.

As far as the barrel/wedge interface is concerned (Figure 33a), the contact pressure attains its peak value (≈425 MPa) at ≈30 mm, that is close to the terminal part of the wedge (see Figure 28a). This effect is due to the differential angle, which (1) avoids the negative pinching effect at the tip of the wedge but (2) reduces the contact area between the barrel and wedge, transmitting high values of pressure to the cable and, consequently, (3) localizes, as already remarked, the contact pressure and shear.

#### 4.2.2. Optimized Anchorages

**Figure 34:**
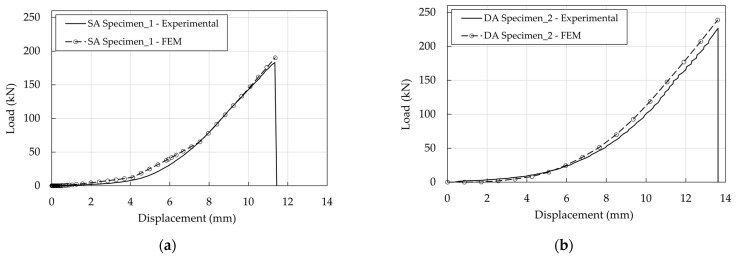
Optimized anchorages. Experimental and numerical force-displacement curves: (**a**) SA anchorage; (**b**) DA anchorage.

For both SA and DA anchorages, the pressure-overclosure relationship at the barrel/wedge interfaces (Figure 24b) was calibrated assuming, as for the traditional anchorage, a friction coefficient (μ) equal to 0.25; in terms of the force-displacement curve (Figure 34a,b) the numerical results fit well the experimental ones.

**Figure 35:**
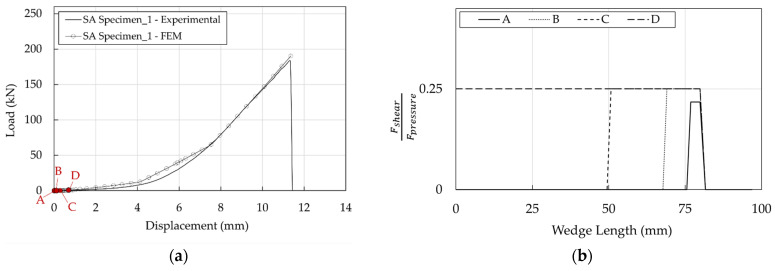
SA anchorage. Barrel/wedge interface: (**a**) Contact pressure; (**b**) Stress-resultant ratios.

The good performance of the system is due to the slippage of the wedges starting from a low value of the force. Considering, for example, the SA anchorage at the four states defined in Table 4 and reported in Figure 35a, the $\frac{F_{\mathrm{shear}}}{F_{\mathrm{pressure}}}$ profiles (Figure 35b) denote that, starting from point B, the value of 0.25 is reached.

**Figure 36:**
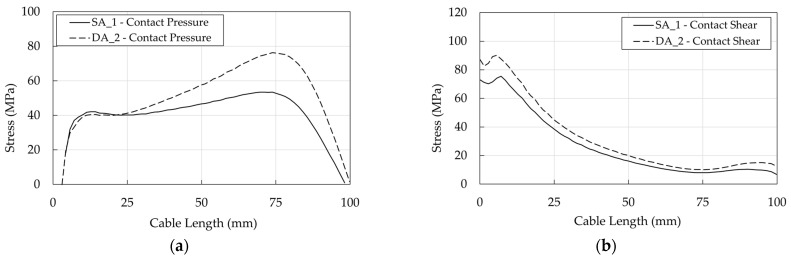
Optimized anchorages. Stress in the resin/cable interface: (**a**) Contact pressure; (**b**) Shear stress.

**Figure 37:**
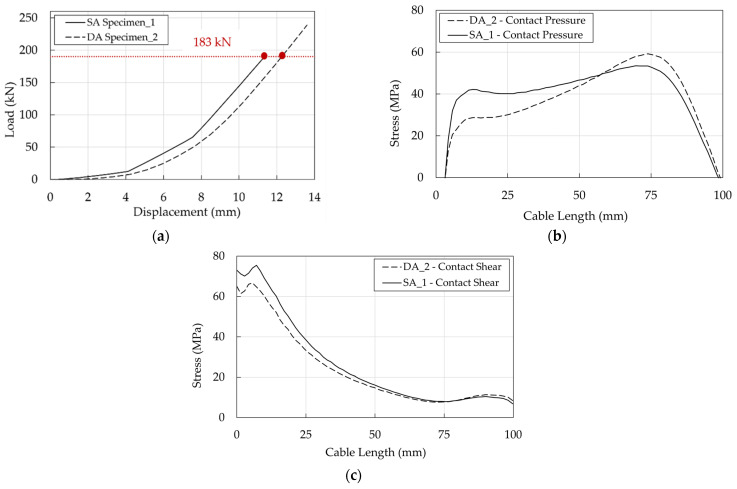
Optimized anchorages. Stress in the resin/cable interface. Numerical results at the same level of axial load (183 kN): (**a**) Force-displacement curves; (**b**) Contact pressure profiles; (**c**) Shear stress.

DA stresses in the resin/cable interface are higher than in the SA (Figure 36a,b), but it has to be considered that the ultimate load of DA (222 kN) is greater than that of SA (183 kN): their ratio is ≈1.2. To provide a more immediate comparison, stresses are reported in Figure 37b,c for a force equal to 183 kN which coincides (Figure 37a) with the failure load of the SA Specimen_1: (1) the shear stress of DA anchorage is closer but lower than the SA and (2) at 10 mm, where the shear has a peak, the contact pressure of the DA is satisfactorily ≈0.7 times lower than that of the SA.

**Figure 38:**
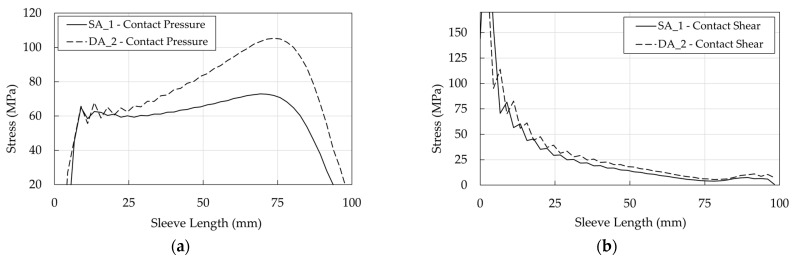
Optimized anchorages. Stress in the wedge/sleeve interface: (**a**) Contact pressure; (**b**) Shear stress.

It is worth noticing that for both optimized anchorages, differently from stress distribution in the traditional anchorage (Figure 31, Figure 32 and Figure 33), the pressure and shear peaks are shifted, reducing the local stress intensity. Previous evidence is remarked in Figure 38, concerning the wedge/sleeve interface: (1) the maximum contact pressure in the sleeve (Figure 38a) occurs at ≈75 mm for both the SA and DA, where the shear stress (≈7 MPa) is close to the minimum value (Figure 38b); (2) the shear stress reaches the highest values at ≈5 mm where the contact pressure is negligible.

**Figure 39:**
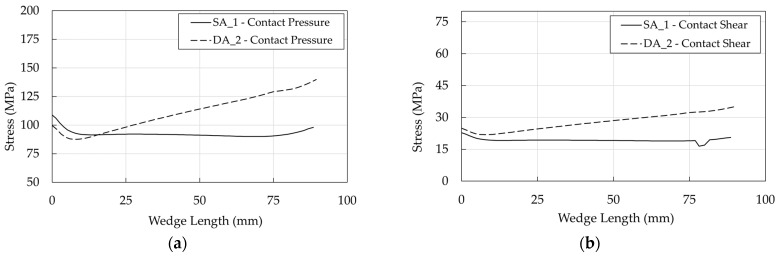
Optimized anchorages. Stress in the barrel/wedge interface: (**a**) Contact pressure; (**b**) Shear stress.

Further on, as far as barrel/wedge is concerned, the pressure and shear profiles (Figure 39a,b) turn out to be almost flat in the SA anchorage, while they exhibit a maximum toward the free end of the anchorage in the DA, where, according to the geometrical model (Figure 14b), only a portion of the wedges is in contact with the barrel. DA pressure and shear stresses reach higher values than the SA, but it should be remarked that stress curves refer to the ultimate condition, corresponding to which, the DA force is higher.

## 5. Conclusions

Through the years, research efforts have made the use of FRP cables a reliable solution within prestressing systems for civil engineering applications. Given the recent advances in the field, the authors of this paper investigated an optimized split-wedge anchorage, for ϕ = 12 mm PCFRP cables, characterized by double-angle (DA) wedges that, differently than single-angle wedges (SA), are shaped according to two different tilting angles. The carried out experimental campaign proved the system efficiency, which reached 100% for one DA specimen, and stimulated the numerical studies presented here which aimed to assess the benefits related to SA and DA systems in terms of contact stresses at the anchorage interfaces.

Preliminarily, the simulation of the tensile test on a traditional anchorage was presented using the ϕ = 12 mm PCFRP cable. It started to slip at ≈58 kN, which corresponds to ≈20% of its tensile capacity. The numerical results satisfactorily matched the peak force of the system and highlighted that wedges remain in the stick phase during the whole loading, triggering the slippage of the cable.

The SA and DA were also numerically studied. The profiles of resultant ratios at the barrel/wedge interface of the SA anchorage confirm the correct behavior of the system, as the wedges start to slip at low values of load. Pressure and shear in the cable, provided at the ultimate condition, generally denoted noncoincident peak values. This result is highlighted by the stress comparison at a fixed value of load of 183 kN, where the DA satisfactorily exhibits a pressure ≈0.7 times lower than that of the SA model in the loading end of the cable. The peak shifting can be considered the main reason of good performance of the proposed systems.

## Figures and Tables

**Table 1:** FEM model: adopted isotropic mechanical properties.

Material	E (GPa)	ν
Steel (Barrel/Wedge)	220	0.3
Aluminum (Sleeve)	70	0.3
Resin	3	0.3

**Table 2:** FEM model: adopted orthotropic mechanical properties.

Material	E_1_ = E_2_	E_3_	G	ν_12_ = ν_21_	ν_13_ = ν_23_
PCFRP	10	160	5	0.3	0.06

**Table 3:** (**a**) Traditional anchorage: adopted contact properties; (**b**) Optimized anchorage: adopted contact properties.

Interface	Direction	Adopted Strategy
(**a**)		
Barrel/Wedge	Normal	Soft (see Figure 24a)
	Tangential	Coulomb (μ = 0.25)
Wedges/Sleeve	Normal	Hard
	Tangential	Rough
Sleeve/Resin	Normal	Hard
	Tangential	Rough
Resin/Cable	Normal	Hard
	Tangential	Coulomb (μ = 0.70)
(**b**)		
Barrel/Wedge	Normal	Soft (see Figure 24b)
	Tangential	Coulomb (μ = 0.25)
Wedges/Sleeve	Normal	Hard
	Tangential	Rough
Sleeve/Resin	Normal	Hard contact
	Tangential	Rough
Resin/Cable	Normal	Hard contact
	Tangential	Rough

**Table 4:** SA anchorage. Initial levels of load.

Point	Force (kN)	Displacement (mm)
A	5.2 × 10^−5^	0.02
B	1 × 10^−2^	0.11
C	0.08	0.29
D	0.70	0.69

## Data Availability

Not applicable.
